# Anthraquinones Extract from *Morinda angustifolia* Roxb. Root Alleviates Hepatic Injury Induced by Carbon Tetrachloride through Inhibition of Hepatic Oxidative Stress

**DOI:** 10.1155/2020/9861571

**Published:** 2020-03-30

**Authors:** Rui-rong Chen, Juan Liu, Zhe Chen, Wen-jun Cai, Xiu-fen Li, Chuan-li Lu

**Affiliations:** ^1^Guangdong Key Lab of Sugarcane Improvement & Biorefinery, Guangdong Bioengineering Institute (Guangzhou Sugarcane Industry Research Institute), Guangdong Academy of Sciences, Guangzhou 510316, China; ^2^College of Food Science and Technology, Yunnan Agricultural University, Kunming 650201, China; ^3^Xishuangbanna Tropical Botanical Garden, Chinese Academy of Sciences, Mengla 666303, China

## Abstract

In Southwestern China, the root of *Morinda angustifolia* Roxb. has been employed as a folk medicine for treating various types of hepatitis and jaundice. The purpose of this study was to evaluate the hepatoprotective effects of anthraquinones extract from *M*. *angustifolia* root (AEMA) in carbon tetrachloride- (CCl_4_-) induced liver injury in mice and identify the main bioactive components. Results indicated that AEMA pretreatment could significantly, in a dose-dependent manner, attenuate the increased levels of ALT and AST in mice serum induced by CCl_4_. At doses of 100 and 200 mg/kg, AEMA exhibited significant suppression of the elevated hepatic levels of malondialdehyde (MDA), as well as marked upregulatory effects on the activities of antioxidant enzymes including superoxide dismutase (SOD) and glutathione peroxidase (GSH-Px) in mice exposed to CCl_4_. However, AEMA treatment had no effect on the antioxidant enzyme catalase (CAT) or the nonenzymatic antioxidant glutathione (GSH). Furthermore, two anthraquinone constituents were isolated from AEMA and identified as soranjidiol and rubiadin-3-methyl ether. Soranjidiol exhibited similar protective effects to those of AEMA on liver damage induced by CCl_4_. Overall, our research clearly demonstrated the hepatoprotective effects of the AEMA, and anthraquinones, particularly soranjidiol, should be considered as the main hepatoprotective principles of *M*. *angustifolia*. In addition, the underlying mechanism may be, at least in part, related to its antioxidant properties.

## 1. Introduction

Liver is the largest and most metabolically active organ in an organism responsible for the detoxification and deposition of endogenous and exogenous substances [1]. Many factors, such as virus infection, improper use of drugs, excessive intake of alcohol, and ingestion of toxic food, could cause acute or chronic liver injury [2]. Furthermore, the sustained liver injury could induce the liver fibrosis, which is the scarring process resulting from those various chronic liver diseases [3], and will lead to serious irreversible cirrhosis and liver damage if the fibrosis is not well controlled [4].

*Morinda angustifolia* Roxb. is a resourceful perennial undershrub or small tree, which is widely distributed in the southwestern mountainous areas of China and nearby countries including India, Nepal, Bhutan, Myanmar, Bangladesh, and Laos [5] and has been used for making yellow dyestuff and food condiment, as well as folk medicine [6, 7]. For instance, in northern Thailand, the plant is used for treatment of digestive system disorders and obstetric diseases [8], while in India, its root extract is used by women after child birth and for treating leucorrhoea with local names “La phoot” and “Aachu gash” [9, 10]. In Bangladesh traditional medical practice, *M*. *angustifolia* has been used for the treatment of urethritis, abdominal tumors, elephantiasis, urinary diseases, insect bites, and fever, with local names “Daru haridra” and “Rong gach” [11, 12]. In Southwestern China, the root of *M*. *angustifolia* has been employed in Dai traditional medicine as a herbal medicine for treating cold, wounds, and inflammation as well as various types of hepatitis and jaundice [7, 13, 14].

Phytochemical investigations have demonstrated that the principal color components isolated from *M*. *angustifolia* roots were anthraquinones such as morindone, aloe-emodin, emodin, rhein, morindonin [6, 15], lucidin-*ω*-butyl ether, 3-*O*-*β*-primeveroside, damnacanthol, 1,3-dihydroxy-2-methylanthraquinone, 1,8-dihydroxy-2-methyl-3,7-dimethoxyanthraquinone, and lucidin-*ω*-ethyl ether [7]. Among those components, 1,8-dihydroxy-2-methyl-3,7-dimethoxy-anthraquinone has been demonstrated to possess significant antimicrobial activity against *Bacillus subtilis*, *Escherichia coli*, *Micrococcus luteus*, *Sarcina lutea*, *Candida albicans*, and *Saccharomyces sake* [7]. Furthermore, the methanol extracts of root and leaves of *M. angustifolia* showed a significant activity in the antipyretic test, which verified the traditional use of the plant in the treatment of febrile condition [12]. In addition, the *in vitro* membrane stabilization and thrombolytic activities of these extracts have also been demonstrated [11].

As mentioned above, *M*. *angustifolia* possesses a well potential for medicinal and healthy products development. However, the phytochemical and bioactive research associated with this plant are very limited. Herein, the *in vivo* hepatoprotective activity of *M. angustifolia* root in CCl_4_-induced liver injury mice model and the potential underlying effective components were reported in present research.

## 2. Materials and Methods

### 2.1. Plant Material

The roots of *Morinda angustifolia*, collected from Menglun Town, Xishuangbanna Autonomous District, Yunnan, China, in November of 2017, were authenticated by Chun-Fen Xiao (Xishuangbanna Tropical Botanical Garden, Chinese Academy of Sciences), and a voucher specimen (No. *M*. *angustifolia*-201711, ML) has been deposited in Bioengineering Laboratory, Guangdong Bioengineering Institute, Guangdong Academy of Sciences.

### 2.2. Chemicals and Reagents

CCl_4_ was purchased from Damao Chemical Industry Co. Ltd. (Tianjin, China). Commercial kits used for measuring the activities of SOD, CAT, and GSH-Px and the levels of GSH, MDA, and protein were all purchased from Nanjing Jiancheng Bioengineering Institute (Jiangsu, China). Hematoxylin-eosin staining (H&E) kit was purchased from Shanghai Lanji Technology Development Co. Ltd. (Shanghai, China). Phosphate buffered saline (PBS) was obtained from GIBCO BRL (Grand Island, NY, USA). All other chemicals were of analytical grade.

### 2.3. General Instrumental Equipment

^1^H and ^13^C nuclear magnetic response (NMR) spectra were recorded on a Bruker AV-500 NMR spectrometer (Bruker, Billerica, MA, USA). Chemical shifts are shown in *δ* values (ppm) with tetramethylsilane as an internal standard.

### 2.4. Extraction and Isolation

The air-dried and crushed roots of *M*. *angustifolia* (1.5 kg) were twice extracted with 3 L ethanol (95%) using heating reflux method for 3 h. The combined ethanol extracts were evaporated to dryness. Then the extract was dissolved with 1 L methanol, and partitioned with equal volume of petroleum ether (60–90°C) four times. The concentrated petroleum ether layer was dried to afford an extract of yellow dyeing (marked as AEMA, 8.57 g). 5.10 g AEMA was subjected to a silica gel column chromatography (CC, 5.0 × 70 cm), eluted with gradient of dichloromethane-methanol (100 : 0, 99 : 1, 98 : 2, 95 : 5, 90 : 10, and 80 : 20). Based on thin layer chromatography results (TLC), the fractions with similar chemical composition were combined to afford eight fractions (Fra 1–8). Fra 5 (0.195 g) was subjected to a silica gel CC (1.5 × 25 cm), eluted with petroleum ether-ethyl acetate 5 : 1 to afford Fra 5-b (0.125 g), which was subsequently purified using Sephadex LH-20 CC (2.5 × 40 cm) eluted with chloroform-methanol (1 : 1) to obtain compound 1(64.2 mg). Fra 7 (0.661 g) was rechromatographed by a silica gel (3.0 × 40 cm) eluted with petroleum ether-ethyl acetate 3 : 1 to afford Fra 7-d (0.143 g), from which compound 2 (31.1 mg) was separated using recrystallization. Isolated compounds 1 and 2 were identified using the basis of spectroscopic data, and the results showed consistency with previously reported data.

### 2.5. Experimental Animals

Six-week old male ICR mice were obtained from the Laboratory Animal Resource Center of the Kunming Medical University and maintained under controlled temperature (22 ± 2°C), humidity (50 ± 5%), and lighting (12 h light/dark cycle) in the Department of Laboratory Animal, Kunming Medical University (Kunming, China). The animals were fed with a standard laboratory diet and given free access to tap water. All animals received humane care according to the institutional animal care guidelines approved by the Experimental Animal Ethical Committee of Guangdong Bioengineering Institute (Guangzhou Sugarcane Industry Research Institute), Guangdong Academy of Sciences.

### 2.6. Treatment of Animals

After a week of adaptation, animals were randomly divided into eight groups, with 8 animals in each group: (1) vehicle control, (2) CCl_4_ model, (3) CCl_4_ + silymarin (SLM, 100 mg/kg), (4) CCl_4_ + AEMA (200 mg/kg), (5) CCl_4_ + AEMA (100 mg/kg), (6) CCl_4_ + AEMA (50 mg/kg), (7) CCl_4_ + Comp. 1 (10 mg/kg), and (8) CCl_4_ + Comp. 2 (10 mg/kg). SLM, AEMA, and two isolated compounds were dissolved in 0.5% CMC-Na solution. Mice were, respectively, given once a day SLM, AEMA, Comp. 1, or Comp. 2 (intragastric administration) for seven days. Six hours after last samples administration, CCl_4_ (0.2%, dissolved in corn oil) intraperitoneal injection was administered at a dose of 7 ml/kg. Mice in vehicle control group received corn oil. 24 h after CCl_4_ treatment, mice were sacrificed, and then plasma and liver tissues were collected.

### 2.7. Measurement of Biochemical Parameters

Serum was collected from blood samples after centrifugation at 3000g for 15 min at 4°C. Serum levels of ALT and AST were measured by an Automatic Biochemical Analyzer (iMagic-M7, Shenzhen iCubio Biomedical Technology Co. Ltd., Shenzhen, China). Liver tissues were washed with normal saline to remove any blood or blood clots. A part of the liver of each mouse was homogenized with ten times its weight of PBS (0.1 M, pH 7.4) and centrifuged at 2500 rpm for 10 min. After removal of cell debris, the supernatant was collected to assess the levels of MDA, GSH, CAT, SOD, and GSH-Px using commercial kits following the standard procedures. In addition, protein content was determined by the Bradford assay [16].

### 2.8. Histopathological Analysis of Liver

Histopathological observation of liver was performed according to the previously reported method [17]. A portion of liver tissues was fixed in 10% PBS formalin solution and embedded in paraffin. Sections were cut into thickness of 5 *μ*m, stained with H&E dyes, and examined under a Leica DM4000 B microscope (Leica Microsystems Inc., Wetzlar, Germany) at 200× magnification.

### 2.9. Statistical Analyses

Results from the animal experiments are presented as mean ± SD. Statistical differences were determined using one-way analysis of variance using the PASW Statistics 18 software (SPSS Inc., Chicago, IL, USA). *P* < 0.05 was considered significant difference.

## 3. Results

### 3.1. TLC Analysis of AEMA

**Figure 1:**
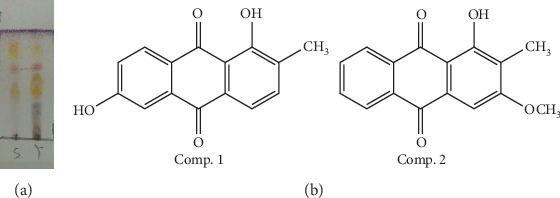
(a) TCL result of AEMA (s) and ethanol extract of *M*. *angustifolia* root (y), eluted by petroleum ether-acetone 9 : 1 (v/v) and colored with 10% H_2_SO_4_ (in ethanol) at 105°C for 3–5 min. (b) Chemical structures of two compounds isolated from *M. angustifolia* roots.

AEMA was obtained as yellow power, while ethanol extract of *M*. *angustifolia* was deep red power. Result of TLC (Figure 1(a)) indicated that the yellow pigments were mainly extracted into petroleum ether fraction when partitioned with petroleum ether and methanol.

### 3.2. Structure Identification of the Isolated Compounds

On the basis of spectroscopic analysis (^1^H NMR and ^13^C NMR) and comparison with the previously reported spectral data [18, 19], Comp. 1 and Comp. 2 were identified as soranjidiol and rubiadin-1-methyl ether, respectively. The chemical structures of two compounds are shown in Figure 1(b), and their spectroscopic data are listed below.

Soranjidiol (Comp. 1, 1,6-dihydroxy-2-methyl-9,10-anthraquinone) was obtained as yellow needle crystal. ^1^H-NMR (CD_3_Cl, 500 MHz) *δ* ppm: 13.14, (s, 1-OH), 8.15 (1H, d, *J* = 8.5 Hz, H-8), 7.65 (1H, d, *J* = 7.5 Hz, H-4), 7.51 (1H, d, *J* = 2.5 Hz, H-5), 7.45 (1H, d, *J* = 7.5 Hz, H-3), 7.14 (1H, dd, *J* = 2.5 and 8.5 Hz, H-7), 2.23 (3H, s, 2-CH_3_). ^13^C-NMR (CD_3_Cl, 125 MHz) *δ* ppm: 188.17 (C-9), 183.45 (C-10), 163.50 (C-6), 160.84 (C-1), 136.59 (C-3), 135.97 (C-10a), 135.27 (C-2), 131.58 (C-4a), 130.00 (C-8), 125.70 (C-7), 121.60 (C-8a), 119.20 (C-4), 115.22 (C-9a), 112.96 (C-5), 16.23 (2-CH_3_).

Rubiadin-3-methyl ether (Comp. 2) was obtained as yellow amorphous power. ^1^H-NMR (CD_3_Cl, 500 MHz) *δ* ppm: 8.21 (1H, dd, *J* = 1.0 and 7.5 Hz, H-8), 8.12 (1H, dd, *J* = 1.0 and 7.5 Hz, H-5), 7.72 (1H, ddd, *J* = 1.5, 7.5 and 7.5 Hz, H-7), 7.66 (1H, ddd, *J* = 1.0, 7.5 and 7.5 Hz, H-6), 7.40 (1H, s, H-4), 3.84 (3H, s, 3-OCH_3_), 2.22 (3H, s, 2-CH_3_). ^13^C-NMR (CD_3_Cl, 125 MHz) *δ* ppm: 185.07 (C-9), 182.98 (C-10), 161.65 (C-3), 161.06 (C-1), 134.97 (C-8a), 134.29 (C-7), 133.88 (C-6), 132.92 (C-10a), 132.44 (C-4A), 127.41 (C-8), 127.09 (C-5), 126.32 (C-2), 118.54 (C-9a), 109.41 (C-4), 60.92 (3-OCH3), 8.95 (2-CH3).

### 3.3. Inhibitory Effects of AEMA and Two Isolated Compounds on the Serum AST and ALT Levels of CCl_4_-Induced Mice

**Figure 2:**
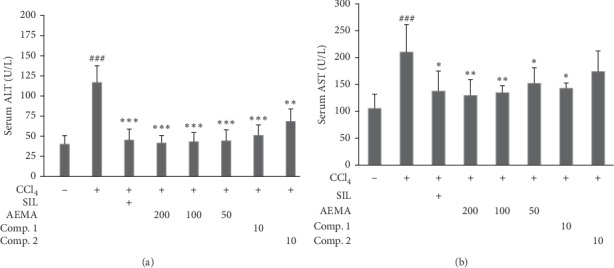
Effects of AEMA and two isolated compounds on the levels of liver enzymes in serum. The mice were treated orally, once daily, with water, silymarin, AEMA at different doses, Comp. 1, or Comp. 2 for seven days. Six hours after the last samples administration, the mice received CCl_4_ (0.2%, 7 ml/kg, i.p.). Control group were treated with vehicle and received corn oil instead of CCl_4_. Values were expressed as mean ± SD (*n* = 8). ### means *p* < 0.001*vs*. control group, while ^*∗*^, ^*∗∗*^, and ^*∗∗∗*^ mean *p* < 0.05, 0.01, and 0.001, respectively, vs. CCl_4_-treated group.

The effects of AEMA and two isolated components on increased activities of serum ALT and AST in CCl_4_-induced liver injury mice are shown in Figure 2. Compared with the normal group, the serum ALT and AST activities of mice in CCl_4_-treated group were significantly elevated (*P* < 0.001). Pretreated by AEMA, the elevated levels of serum ALT and AST in CCl_4_-induced mice were significantly decreased in a dose-dependent manner (*P* < 0.05). Silymarin as the positive control could also significantly reduce both ALT and AST activities (*P* < 0.001 and 0.05, respectively). In addition, pretreatment with Comp. 1 (at dose of 10 mg/kg) also exhibits markedly inhibitory effects on CCl_4_-induced elevation of serum ALT and AST activities (*P* < 0.001 and 0.05, respectively). However, Comp. 2 pretreatment (at dose of 10 mg/kg) just alleviates the serum ALT activity, but has no effect on AST activity for CCl_4_-treated mice.

### 3.4. Suppression Effects of AEMA and Its Two Main Components on Liver MDA, GSH, CAT, SOD, and GSH-Px of Experimental Mice

**Figure 3:**
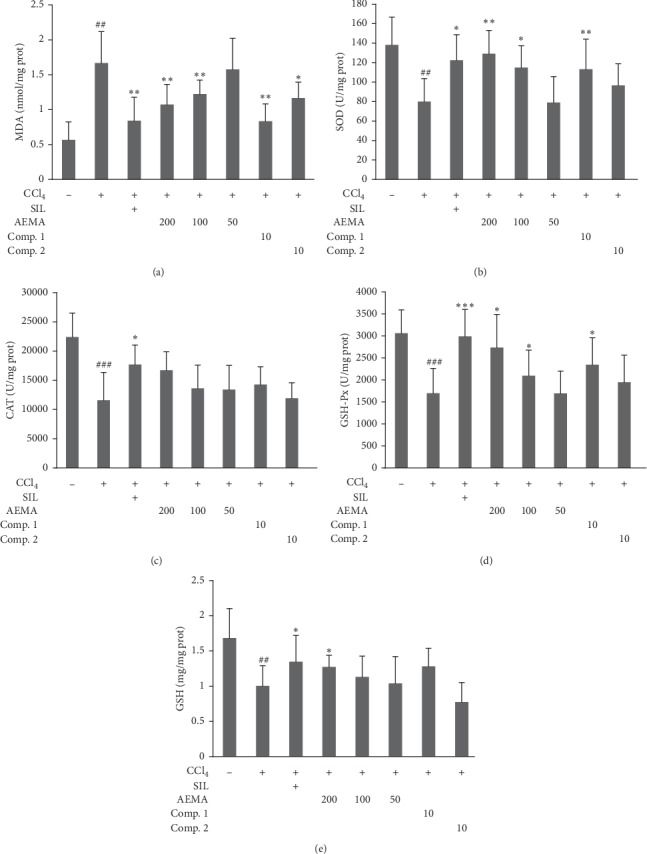
Effects of AEMA and two isolated compounds on hepatic MDA level and antioxidant enzymes. The mice were treated orally, once daily, with water, silymarin, AEMA at different doses, Comp. 1, or Comp. 2 for seven days. Six hours after the last samples administration, the mice received CCl_4_ (0.2%, 7 ml/kg, i.p.). Control group were treated with vehicle and received corn oil instead of CCl_4_. Values were expressed as mean ± SD (*n* = 8). ## and ### mean, respectively, *p* < 0.01 and 0.001 *vs*. control group, while ^*∗*^, ^*∗∗*^, and ^*∗∗∗*^ mean *p* < 0.05, 0.01, and 0.001, respectively, vs. CCl_4_-treated group.

The results of hepatic biochemical indicators detecting are presented in Figure 3. Compared with the normal group, CCl_4_ treatment markedly induced an increased content of MDA in the liver (*P* < 0.01, Figure 3(a)) and decreases in hepatic SOD (*P* < 0.01, Figure 3(b)), CAT (*P* < 0.001, Figure 3(c)), and GSH-Px (*P* < 0.001, Figure 3(d)) activities, but did not affect hepatic level of GSH (*P* < 0.05, Figure 3(e)). Pretreated by AEMA at doses of 200 and 100 mg/kg, the increased hepatic level of MDA was, respectively, decreased by 35.9% and 26.9%, compared with CCl_4_-treated group (Figure 3(a)). In addition, the activities of hepatic SOD and GSH-Px in AEMA pretreatment group were obviously recovered (Figures 3(b) and 3(c)). However, there is no significant difference in CAT activity or GSH level between AEMA treatment group and CCl_4_-treated group (Figures 3(d) and 3(e)). Interestingly, Comp. 1 (at 10 mg/kg) exhibits similar effects on hepatic parameters to those of AEMA (at 200 mg/kg), which significantly inhibits the MDA generation (Figure 3(a)), as well as increasing the activity of SOD (Figure 3(b)) and GSH-Px (Figure 3(c)) in liver of mice exposed to CCl_4_, but has no effect on liver GSH level and CAT activity (Figures 3(d) and 3(e)). However, Comp. 2 just shows a marked effect on MDA generation in liver of mice exposed to CCl_4_ (Figure 3(a)).

### 3.5. Histopathological Observation

**Figure 4:**
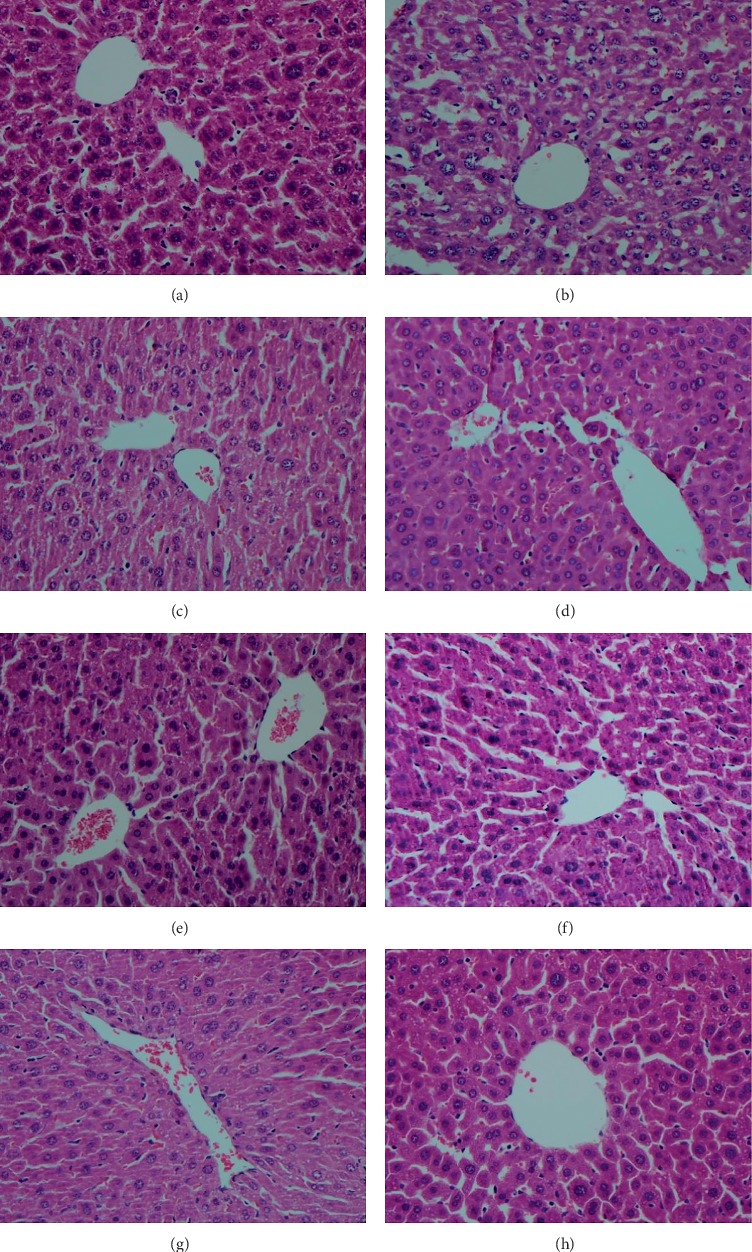
Histopathology of hepatic tissue section (200×): (a) vehicle control group, (b) CCl4 model group, (c) CCl4 + silymarin (100 mg/kg) group, (d) CCl4 + AEMA (200 mg/kg) group, (e) CCl4 + AEMA (100 mg/kg) group, (f) CCl4 + AEMA (50 mg/kg) group, (g) CCl4 + Comp. 1 (10 mg/kg) group, (h) CCl4 + Comp. 2 (10 mg/kg) group.

The histopathological changes observed in the liver tissues of different groups are illustrated in Figure 4. As shown in Figure 4(a), the histology of liver sections from normal group mice displayed well-preserved hepatocytes with prominent nucleus, nucleolus, uniform cytoplasm, and radial arrangement around the central vein. However, the liver sections from mice exposed to CCl_4_ showed severe cellular degeneration, hepatocyte necrosis, and loss of cellular boundaries (Figure 4(b)), suggesting that CCl_4_-induced liver injury model was successfully established. On the other hand, both the treatments with silymarin and different doses of AEMA could effectively attenuate hepatocellular damage as reflected by the reduction of necrotic areas and inflammatory cell infiltration induced by CCl_4_ (Figures 4(c)–4(f)). In addition, pretreatment with both isolated compounds also ameliorated the hepatocellular injury induced by CCl_4_ (Figures 4(g) and 4(h)).

## 4. Discussion

The root of *M*. *angustifolia* is a common folk medicinal material in Dai traditional medicine in Southwestern China and is widely used for treating various types of hepatitis and jaundice. However, to the best of our knowledge, there are few reports about the live protective effect of *M*. *angustifolia*. In the present study, the *in vivo* hepatoprotective activities of yellow pigments extracted from *M*. *angustifolia* root and its two derived constituents were demonstrated in a liver injury mice model induced by CCl_4_.

We found that CCl_4_ treatment induced severe hepatocellular degeneration, necrosis, and liver cells with no regular arrangement around the central vein. However, the pathologic degree of injury was reduced after AEMA and Comp. 1 treatment, suggesting they played a positive role in protecting against liver damage.

ALT and AST have been widely accepted as two major biomarkers to assess the hepatic injury [20]. ALT is a cytosolic enzyme that is normally distributed in the hepatocytes, while AST is primarily localized in mitochondria and cytoplasm of liver cells; thus, the increased serum levels of ALT and AST, respectively, indicated that the damage of hepatocytes reached the level of cell membranes and organelle [21, 22]. In this study, we found that pretreatments with AEMA (50–200 mg/kg) could markedly downregulate the elevated serum levels of ALT and AST in mice exposed to CCl_4_, which means that AEMA possesses the potential protective effect against the hepatotoxicity induced by CCl_4_ in mice. This result is consistent with the findings that *Morinda citrifolia* juice and ethanol extract of *Morinda pubescens* fruit reduced the increased AST and ALT activities [23, 24]. In addition, the isolated anthraquinone, soranjidiol, exhibited suppression effects on elevated ALT and AST activities similar to those of AEMA, which suggested that soranjidiol should be one of the main hepatoprotective constituents of *M*. *angustifolia*.

In the liver, CCl_4_ is rapidly metabolized to trichloromethyl (•CCl_3_), a bioactive free radical, which could attack and destroy polyunsaturated fatty acids and increase lipid peroxidation in cellular and organelle membranes, resulting in necrosis and apoptosis of hepatocytes [25, 26]. Malondialdehyde (MDA) is a final product of polyunsaturated fatty acids peroxidation, and its concentration reflects the severity of lipid peroxidation [27]. In our study, MDA levels in the liver of CCl_4_-exposed mice were markedly elevated, which suggested that the lipid peroxidation and oxidative stress in liver increased. Pretreatment with AEMA (200 and 100 mg/kg) and its two isolated compounds (10 mg/kg) significantly inhibited the over-formation of MDA in the liver induced by CCl_4_, which suggest that AEMA and its two components (particularly Comp. 1) might have preventive effects on liver injury by reducing the formation of lipid peroxidation *in vivo*. These results are consistent with the findings that polysaccharides from *Mesona blumes* induced reduction of the increased MDA [26].

SOD, CAT, and GSH-Px are the most important antioxidant enzymes in mammalian system, which can inhibit the formation of free radicals and serve as indicators for the production of reactive oxygen species [28]. Among these antioxidant enzymes, SOD can catalyze the dismutation of superoxide anions into hydrogen peroxide (H_2_O_2_) and oxygen (O_2_) [29], and CAT can catalyze the breakdown of H_2_O_2_ into O_2_ and water [30], while GSH-Px catalyzes the reduction of H_2_O_2_ and other hydroperoxides into nontoxic compounds with GSH as the electron donor and terminates the chain reaction of lipid peroxidation by removing lipid hydroperoxides from the cell membrane [31, 32]. Results of the present study obviously showed that treatment with CCl_4_ could significantly induce the decreases in the activities of hepatic SOD, CAT, and GSH-Px compared with the normal group mice. However, pretreatment with AEMA could markedly elevate the activities of SOD and GSH-Px in the liver of mice treated with CCl_4_. In addition, Comp. 1 also exhibits ameliorative effects on hepatic parameters similar to those of AEMA, but Comp. 2 did not show any effect on these hepatic antioxidant enzymes. Similar results in other researches also confirmed that natural anthraquinones, e.g., chrysophanol [33], emodin [34], and rhein [35], played a protective role in the reduction of oxidative stress and upregulated SOD and GSH-Px levels in different liver injury animal models. Thus, it is possible that the mechanism of AEMA and Comp. 1 attenuates CCl_4_-induced liver injury in mice and may be related to their antioxidant properties. Furthermore, the antioxidant enzymes including SOD and GSH-Px are mainly regulated by the nuclear factor erythroid 2-related factor 2 (Nrf2) signaling pathway, which is an important regulator of the antioxidant defense system [36, 37]. However, more studies are needed to reveal whether Nrf2 pathway played a key role in liver protective activities of the AEMA and Comp. 1.

## 5. Conclusion

In conclusion, the results of the present study, for the first time, clearly demonstrated that AEMA possesses a significant protective effect against liver injury in CCl_4_-treated mice. The underlying mechanism for hepatoprotective effect of AEMA may be, at least in part, related to its regulatory effects on liver oxidative stress. Comp. 1 showed similar protective effects to those of AEMA on liver injury induced by CCl_4_ and should be considered as one of the main hepatoprotective constituents of *M*. *angustifolia*. These findings suggested that AEMA and Comp. 1 might be explored as potential hepatoprotective drugs.

## Data Availability

All data used to support the findings of this study are available from the corresponding author upon request.
